# Vaginal Mesh Kits for Pelvic Organ Prolapse, Friend or Foe: A Comprehensive Review

**DOI:** 10.1100/tsw.2009.19

**Published:** 2009-03-01

**Authors:** Robert D. Moore, John R. Miklos

**Affiliations:** Atlanta Urogynecology Associates, Northside Hospital, Atlanta, GA, USA

**Keywords:** cystocele, rectocele, vaginal mesh, mesh complications, prolapse, Perigee, Prolift

## Abstract

Graft use in vaginal prolapse surgery has become more common secondary to high failure rates seen with traditional repairs. Mesh has been shown to be successful when suspending the upper portion of the vagina with sacralcolpopexy and its use vaginally is in an attempt to reproduce those results seen from the more invasive abdominal approach. A recent Cochrane review has supported its use in the anterior compartment vaginally as lower failure rates have been shown. Vaginal mesh “kits” have been developed in an attempt to make these surgeries less invasive, more standardized, and easier to perform. One of the problems that does seem to be emerging is the thought that, just because these procedures are now being produced in “kits”, they can be completed by *any* surgeon. This may not hold true, as these are still advanced techniques that require advanced pelvic surgery skills and, therefore, it is up to surgeons to also understand this and the limitations of these procedures. The current paper reviews the history of the development of mesh kits, the techniques utilized, and the data that have been published to date on results and complications, and recommendations on how to avoid these complications. Although initial studies are encouraging, more will need to be completed prior to the recommendations of general use of these kits in all prolapse patients. We need to have further investigation on proper patient selection, we must continue research on graft composition, and we must continue to develop techniques to minimize complications of needle passage or mesh placement. Even after we gain this knowledge, it will still require advanced surgical skills to complete these types of surgeries, and to help minimize complications and maximize results.

## INTRODUCTION

Pelvic organ prolapse (POP) is a significant health issue in females worldwide[1,2]. There are approximately 250,000 procedures performed annually in the U.S. for POP, with many women having to undergo repeat surgery due to failure of previous procedures[2]. Traditional vaginal repair of anterior, posterior, or apical prolapse that utilizes the patient's own tissue is a compensatory procedure that has reported high failure rates, and can result in vaginal shortening and/or constriction. Additionally, plication or colporrhaphy techniques on the anterior or posterior walls address only midline defects and attach poor-quality tissue to poor quality tissue, under tension, which most likely contributes to the high failure rate associated with these types of repair. In the anterior compartment, paravaginal repair completed abdominally or laparoscopically is a more anatomic procedure; however, it still relies on reattaching the patient's own native tissue, which has already failed, back out to the arcus tendineus (white line) and has never been proven to be more effective in long-term cure rates than vaginal anterior repair[3]. The longevity of vault procedures that utilize the patient's native tissue, such as the uterosacral ligaments, has also been questioned because the vault is suspended to ligaments that have already failed and, therefore, may not have the inherent strength to maintain support.

Purists will say that if the anatomy of the defect is repaired properly and perfectly with the patient's own tissues, then the repair should withstand the forces that are produced into the pelvis on a daily basis and the cure rates should be adequate. However, it is a well-known fact that, across the board, 29% of patients who undergo traditional repairs will have failure and require a repeat surgery[2]. This may be due to poor surgical technique of the procedure, inadequacy of the procedure to handle more advanced prolapse in some patients, or may just be due to the fact that patients with prolapse may have inherently and genetically weak tissue with defects in collagen and smooth muscle structure and strength[4,5]. It is felt that even if the anatomy is repaired perfectly, this weak endogenous tissue will deteriorate rapidly, causing recurrent fascial or connective tissue separation from its connection in the pelvis.

There are two major factors that come into play in any pelvic floor repair: the tissues being repaired, i.e., the pubocervical and rectovaginal fascia, and the attachment of this fascia to fixed structures in the pelvis at all three levels of pelvic floor support, i.e., distal, lateral, and apical. The fixed distal attachment is at the level of the perineal body. The lateral attachment is at the arcus tendineus fascia pelvi (the white line) that is a tendinous line that forms where the levator ani muscles are attached to the obturator internus muscle in the pelvis and runs from the pubic symphasis distally to the ischial spines apically. Lastly, the cardinal/uterosacral complex forms the apical attachment. Failure can occur because the central fascia support of the organs gives way or, more likely, when its attachments to these fixed structures in the pelvis give way. One of the major problems is, however, that no endogenous tissue repair ever returns connective tissue elements to baseline tensile strength. The literature on hernia repair for the abdominal wall suggests that graft augmentation greatly enhances success rates over time. Graft repairs to compensate for weak native tissues have reduced failure rates from 58 to 23%[6] and, because of this, graft use has become an accepted common practice among general surgeons for abdominal wall hernia repair.

Graft use in vaginal prolapse surgery is still somewhat controversial; however, it has been proven to be very effective and has become a standard of care in the treatment of severe apical prolapse. Abdominal Y-mesh sacralcolpopexy has the highest cure rates in the literature for vault prolapse and the benefit of utilizing mesh in the repair seems to outweigh the risks[7]. It results in an anatomic repair with minimal tension and does not rely on the patient's weakened tissue to maintain support. In the Y-mesh technique of the sacralcolpopexy, up to 75% of the vagina may be covered with mesh, which helps to support not only the apex, but also the anterior and posterior walls. The mesh support of the apex is the anchor of the entire repair and most likely why this has the highest overall cure rates in the literature for any prolapse repair. However, the sacralcolpopexy is an abdominal procedure that is more invasive than vaginal approaches for prolapse and requires a high skill level, especially with a laparoscopic approach. Therefore, the search has blossomed in recent years for less invasive vaginal procedures to attempt to replicate the higher cure rates seen with abdominal approaches.

With the success of apical graft use, graft augmentation of prolapse repair has been more recently utilized via the vaginal route. Julian first reported on the use of synthetic mesh for cystocele repair in 1996[8] and, more recently, there have been multiple reports of various methods to place grafts via a vaginal approach for cystocele repair[9,10,11,12,13,14,15,16,17,18], which are summarized in Table 1. Graft use to treat the posterior compartment (i.e., rectocele and enterocele) as well as vault prolapse has also been investigated and has shown higher cure rates in many series (Table 2) compared to traditional approaches or historical controls[12,13,15,19,20,21,22,23]. Although improved cure rates compared to traditional repairs

**TABLE 1**
**Anterior Prolapse Surgery (Synthetic Mesh)**

| Study | Graft/Mesh | No. of Patients | Follow-Up (Months) | Anatomic Cure | Comments |
|---|---|---|---|---|---|
| Julian[8] | Marlex | 12 | 24 | 100% | 25% Mesh erosion |
| Nicita[9] | Marlex | 44 | 14 | 90% | 2.5% Mesh erosion |
| Migliari and Usai[10] | Mixed fiber mesh | 15 | 23.4 | 93% | No mesh-related complications |
| Migliari et al.[11] | Polypropylene | 12 | 20.5 | 100% (Subjective) | No mesh-related complications |
| Sand et al.[12]* | Polyglactin 910 | 80 | 12 | 75% | No mesh-related complications |
| Dwyer and O'Reilly[13] | Polypropylene (Atrium) | 64 | 29 | 94% | 4.6% Mesh erosion, 6% posterior compartment prolapse |
| De Tayrac et al.[14] | Polypropylene | 84 | 24 | 91.6% | 8.3% Mesh erosion |
| Milani et al.[15] | Polypropylene | 32 | 17 | 94% | 13% Mesh erosion, 38% dyspareunia |
| De Tayrac et al.[16] | Polypropylene (Gynemesh) | 63 | 37 | 89.1% | 9.1% Mesh erosion, 5.5% mesh-related pain |
| De Tayrac et al.[21] | Low-weight polypropylene with absorbable film | 143 | 13 | 92.3% | 6.3% Mesh erosion, 12.8% dyspareunia. |
| Hiltunen et al.[18]* | Low-weight polypropylene | 104 | 12 | 93.3 vs. 61.5% (Anterior repair) | 17% Mesh erosion, increase in post-op SUI |
| Sivaslioglu et al.[77]* | Polypropylene (Sofradim) | 90 | 12 | 91 vs. 72% (Anterior repair) | 6.9% Erosion, 4.6% dyspareunia |
| Nieminen et al.[73]* | Low-weight polypropylene | 105 | 24 | 89 vs. 59% (Anterior repair) | 8% Erosion, dyspareunia lower in mesh group |

* Denotes randomized controlled trial.

Table modified from Ridgeway et al.[66].

have been reported, most of these techniques require advanced surgical skills, require large and difficult dissections, and can carry higher morbidity compared to traditional repairs. Many different techniques have been reported on various means to attach the graft in place, as this has been proven to be somewhat difficult, especially in the anterior compartment. The techniques may differ significantly in the dissection, the type of material utilized, the size or shape of the graft, and where the attachment of the graft is made, both laterally and apically. Some series have reported a "tension-free" placement of the graft and define this as not using any sutures to attach the graft in place either laterally or apically[16]. This lack of standardization, as well as the more complex nature of these repairs (and the unknown risks, such as mesh complications, dyspareunia, etc.), resulted in slow acceptance of these techniques to utilize grafts in vaginal prolapse repairs.

**TABLE 2**
**Posterior Prolapse Surgery (Synthetic Mesh)**

| Study | Graft/Mesh | No. of Patients | Follow-Up | Anatomic Cure | Comments |
|---|---|---|---|---|---|
| Watson et al.[19] | Polypropylene | 9 | 29 Months | Functional cure 89% | No mesh-related complications |
| Sand et al.[12]* | Polyglactin 910 | 65 | 24 Months | 90% | No mesh-related complications |
| Dwyer and O'Reilly[13] | Polypropylene | 50 | 29 Months | 100% | 12% Mesh erosion, 1 rv** fistula |
| Lim et al.[20] | Composite polyglactin 910-polypropylene | 90 | 6–12 Weeks, n = 31, to 6 months | 98.9% at 6–12 Weeks, 87.5% at 6 months | 7.8% Mesh erosion at 6–12 weeks, 12.9% at 6 months |
| Milani et al.[15] | Polypropylene (not soft) | 31 | 17 Months | 100% | 6.5% Mesh erosion |
| De Tayrac et al.[21] | Polypropylene to SSL** | 26 | 23 Months | 92.3% | 12% Mesh erosion, 7.7% *de novo* dyspareunia |
| Lim et al.[22] | Composite polyglactin 910-polypropylene | 78 | 36 Months | 78% | 30% Mesh erosion, 27% *de novo* dyspareunia |
| DeTayrac et al.[23] | Polypropylene with hydrophilic absorbable coating | 76 | 12 Months | 97.4% | 6.3% Extrusion, 12% dyspareunia |

* Denotes randomized controlled trial.

** rv, rectal vaginal; SSL, sacrospinous ligament.

Table modified from Ridgeway et al.[66].

## VAGINAL MESH KITS FOR PROLAPSE REPAIR

The benefits of graft use for vaginal prolapse seemed very obvious, especially with the long-term results of mesh sacralcolpopexy for vaginal vault prolapse, and the seemingly higher cure rates seen in some of the initial trials of vaginal mesh placement for treatment of cystocele and rectocele. Although POP repair can be achieved through multiple routes, most surgeons agree that the vaginal approach offers less morbidity and pain, and quicker recovery than abdominal approaches via laparotomy. These features often make surgical correction an option for patients not previously considered candidates for surgical repair. Although the abdominal approach via laparotomy has been associated with higher cure rates, it also carries higher morbidity and increased recovery time. Laparoscopic reconstruction is an option and has shown equivalent cure rates[24]; however, this takes advanced laparoscopic skills, therefore limiting the access for many patients to this approach as well. The challenge becomes achieving the durability of the abdominal approach, while preserving the minimally invasive nature of the vaginal approach. In the past, mesh or graft augmentation had limited use because it was perceived to require specialized surgical skills and extensive dissections that would result in increased patient morbidity, blood loss, and postoperative pain; increased risks of bladder or bowel perforation; and prolonged recovery. The lack of standardized mesh placement may also have contributed to this apprehension, with more than 40 techniques described in the literature for vaginal vault prolapse alone[1].

Recently, vaginal mesh kits for prolapse repair have been developed in an attempt to reproduce the benefits seen with standardized kits that have been developed for treatment of female stress urinary incontinence (SUI), such as the tension-free vaginal tape (TVT) sling procedure (Gynecare, Sommerville, NJ) and subsequent transobturator tape (TOT) sling kits. The TVT sling revolutionized the treatment of female SUI by introducing a much more simple and less invasive approach, and by having all of the necessary equipment, graft, and needles packaged together in one kit. The procedure was taught in a standardized fashion and the results were reproduced in multiple studies throughout the world. It became the standard of care in sling procedures for the treatment of female SUI. Subsequent procedures, such as the TOT sling, utilized this same model and have been just as successful[25,26]. The Apogee/Perigee vaginal mesh kits (American Medical Systems, Minnetonka, MN) were the first kits to be released in the U.S. for treatment of POP in 2004 (Figs. 1 and 2). Subsequently, the Prolift and Avaulta vaginal mesh kits were released by Gynecare (Fig. 3) and Bard Urological (Covington, GA) (Fig. 4), respectively, and offer minor variations in the kits; however, the premise behind all of the procedures is essentially the same, i.e., utilization of a kit for a standardized vaginal approach to treat POP with options to treat all compartments simultaneously, if necessary. This paper will review the procedures, risks, benefits, potential complications, and published clinical results of these modalities.

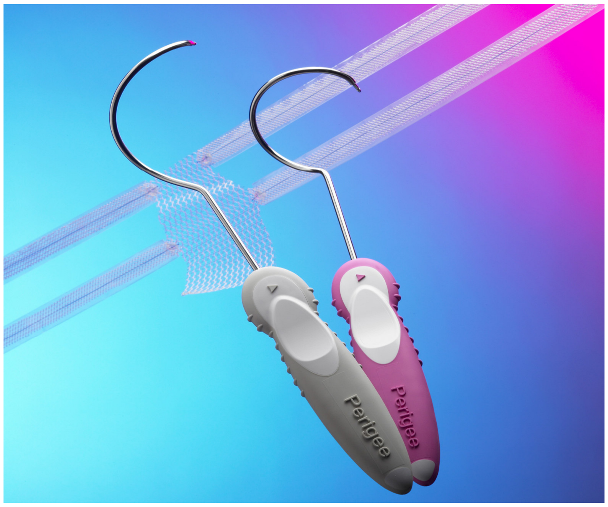

**FIGURE 1**. Perigee System by American Medical Systems.

## VAGINAL MESH KITS FOR THE ANTERIOR COMPARTMENT (CYSTOCELE)

The transobturator space has been shown to be a very safe space for the placement of tension-free tape slings for the treatment of SUI and has simplified the technique of this procedure[27,28,29]. The space has also been recently utilized to secure the anterior arms of a tension-free graft for cystocele repair; however, no apical attachment of the graft was described[16]. The Perigee System (American Medical Systems) was the first commercial kit to be released in the U.S. in 2004 and contains four side-specific, patented, helical needles designed for each anatomic pass through the obturator space to attach a graft to the pelvic side wall in four locations: distally at the level of the bladder neck and apically at the level of the ischial spines. The kit includes a soft, monofilament, macroporous polypropylene mesh (Intepro™) that has four self-fixating, adjustable arms with special connectors to attach to the needles (Fig. 1). The graft arms also feature a tensioning suture designed to maintain the integrity of the mesh arms as they are deployed through the anatomic passages, and during the adjustment of the arms and the midline graft. Recent modifications of the Perigee System include a longer inferior needle to achieve placement of the more apical attachment closer to

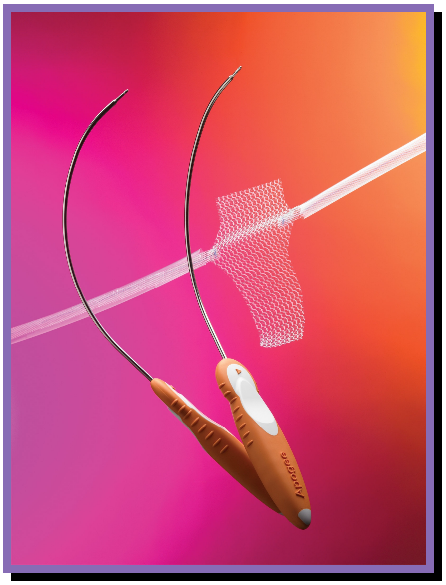

**FIGURE 2.** Apogee by American Medical Systems.

the ischial spine and a mesh (Intepro Lite™) that is 50% less dense and lighter than its predecessor, resulting in a softer mesh with overall less mesh load. The anterior Prolift (Gynecare, Johnson and Johnson) System also uses a Type I macroporous, polypropylene mesh (Gynemesh) with four lateral arms and with similar needle passes through the groin; however, it only uses one curved-shaped needle for each pass (same needle is used for the posterior system as well; Fig. 3) and the mesh itself is 20% larger compared to that in the Perigee System. The anterior Avaulta System (Bard Urological) is the most recent kit to be released and similarly uses just one needle for all passes through the groin (and again the same needle is used for the posterior system), with a suture-capturing device to attach the mesh arms to the needle to try to help simplify the needle attachment to the mesh arms. However, the system utilizes a porcine collagen-coated mesh that is more dense, stiffer, and heavier than both the Intepro Lite and Gynemesh, and may not be considered a Type I mesh because of the coating. The coating was placed on the mesh in an attempt to decrease the initial inflammatory response during healing and to decrease, hopefully, the risk of mesh extrusion; however, this has not been confirmed in any clinical studies to date[30].

## Surgical Procedure and Relevant Anatomy

In the transobturator approach, to place an anterior wall graft (which all three systems utilize), needles are passed through the groin and the obturator space, and penetrate the pelvic side walls to attach the lateral arms of the mesh graft to the pelvic side wall at the level of the bladder neck and ischial spine. The advantage of this approach is that it allows simultaneous treatment of midline, lateral, and apical defects of the pubocervical fascia as the graft is attached from side wall to side wall from the bladder neck up to the ischial spines, with excellent fixation through the fascia of the pelvic side wall. The procedure is typically completed under spinal or general anesthesia. The anterior wall should be infiltrated with a vasoconstrictor solution to help with dissection and minimize blood loss. A small incision (approximately 3 cm) is made starting at the level of the bladder neck and made toward the apex of the vagina or the cervix if the uterus is *in situ*. The incision should be kept to a minimum and not taken all the way up to the apex of the vagina. A deep dissection plane should be sought to keep the vaginal epithelium that overlies the graft thicker to help protect against mesh complications. The dissection is taken out laterally

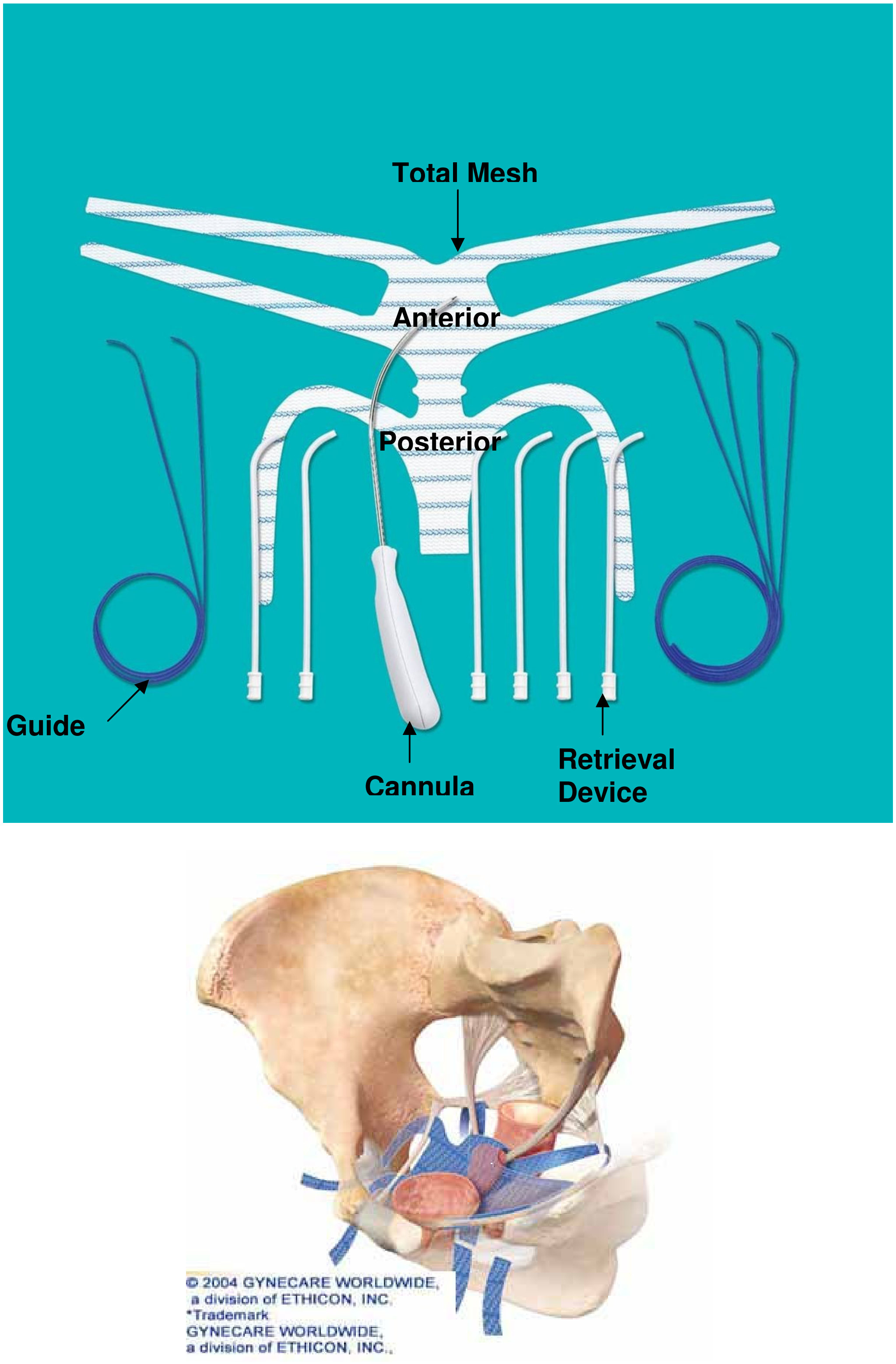

**FIGURE 3.** Prolift System by Gynecare Worldwide, a division of Ethicon, Inc.

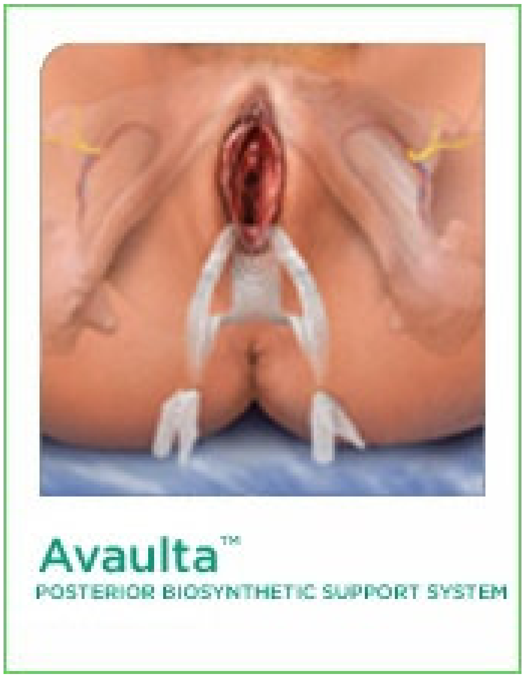

**FIGURE 4.** Avaulta System by Bard Urological.

to the side wall up to the level of the ischial spines, and the bladder should also be dissected apically off the cervix or the vaginal cuff. The needles are then passed through the transobturator space, attached to the lateral arms of the graft, and the arms pulled back through the side walls and the groin incisions (Figs. 5–7). The apical portion of the graft is then tailored to the length of the vagina, sutured to the apex, and the arms then adjusted in a tension-free manner. The final positioning places the mesh under the bladder and attaches it laterally to the arcus tendineus from the bladder neck up to the ischial spine bilaterally (Fig. 8). Minimal vaginal epithelium should be excised secondary to the fact that tissue shrinkage is thought to occur around mesh grafts and the incision should be closed with absorbable sutures. Prior to removing the plastic sheaths of the Perigee System, the corners of the vagina should be checked to ensure that the lateral arms of the graft that are penetrating the side walls are not under tension. If they are found to be under tension, the surgeon's finger should be used to lift up on the arms to loosen them prior to taking the plastic outer sheath off the mesh arms, which will lock the arms in place. The other systems do not use the plastic sheaths that help during adjustment and tensioning; therefore, the surgeon needs to be very careful not to place the arms under tension during the initial placement. Cystoscopy should be completed following the repair to evaluate ureteral integrity and to ensure no mesh penetration into the bladder.

It should be noted that anterior wall mesh placed in the above manner for cystoceles does not treat incontinence and patients will still require a midurethral sling completed at the time of repair. This can be done concomitantly and it is recommended that the anterior wall mesh be placed first and the normal anatomy of the anterior wall be re-established prior to placing the sling. A separate suburethral incision should be made to ensure that the sling stays in position under the midurethra and does not slide up under the other mesh at the bladder neck. A retropubic, or TOT, approach may be utilized to place the sling. If the TOT approach is utilized, the same groin incisions for the superior pass of the anterior wall mesh may also be used to place the TOT sling. Recently, Amrute et al.[67] addressed this issue and modified an anterior wall mesh graft to treat incontinence simultaneously with anterior wall prolapse with one procedure/graft. They utilized an H-shaped graft to support the anterior vault up to the sacrospinous ligaments, the body to support the cystocele, and the distal arms were placed under the midurethra and

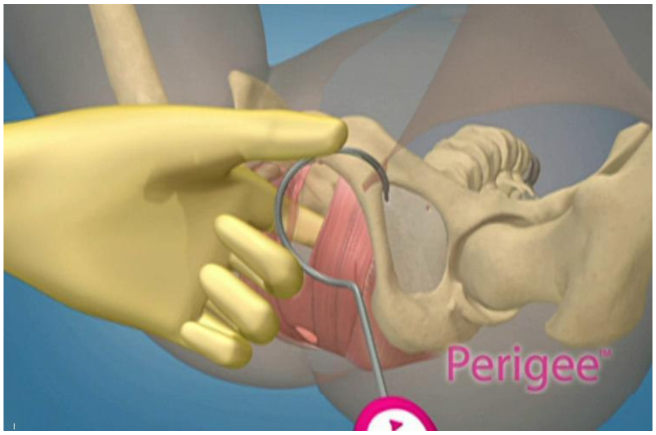

**FIGURE 5.** Superior needle pass of Perigee.

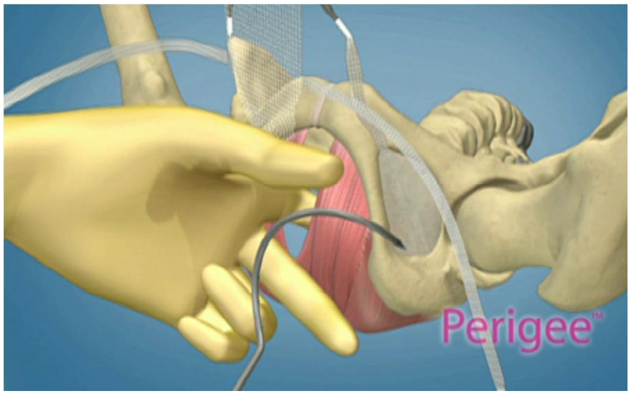

**FIGURE 6.** Inferior/apical needle pass of Perigee.

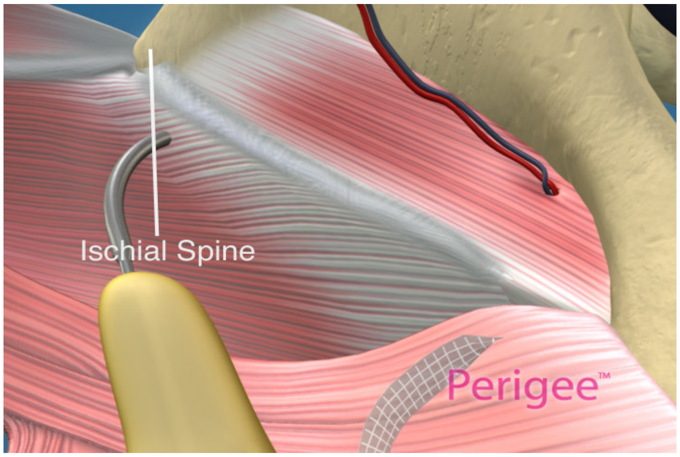

**FIGURE 7**. Apical needle pass of Perigee System through side wall.

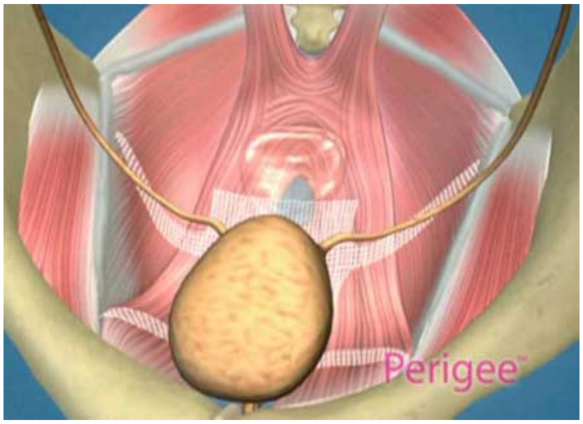

**FIGURE 8.** Final position of mesh under bladder of Perigee System.

anchored retropubically either with bone anchors or with passage of Bioarc™ (American Medical Systems) needles retropubically to treat SUI. They found that by using one graft, they could successfully treat both prolapse and incontinence simultaneously. This procedure, to date, has not been reproduced commercially in a kit.

## GRAFT USE IN THE POSTERIOR COMPARTMENT

Rectocele repair traditionally relies on the plication of attenuated rectovaginal fascia by posterior colporrhaphy or levatorplasty, or the more recent site-specific defect repair initially described by Richardson[31]. The reported success rate of posterior colporrhaphy with plication is only 65–75% on medium-term follow-up (1–2 years), with significant decline after 3 years[32]. According to Cundiff and Fenner in their recent review of the literature on rectocele evaluation and treatment, posterior colporrhaphy is associated with an up to 15% *de novo* rate of dyspareunia, with only modest relief of associated symptoms typically caused by rectoceles[33]. The anatomic cure rate for site-specific defect repair seems to be slightly higher and has been reported between 72 and 85%, with rates of *de novo* dyspareunia for site-specific repair being minimal at 1–3%, with good relief of associated symptoms[33,34,35].

However, again, both types of repairs (plication or site-specific) rely on tissue that has already failed or is weakened; therefore, the use of graft material to augment or support the repair has begun in the posterior compartment with initial studies that show improved cure rates over traditional repairs that utilize biologic grafts[36,37]. However, graft use in either compartment is still limited by a lack of knowledge of the correct material to use and in what surgical setting the grafts may be efficacious. It was thought that graft augmentation of traditional rectocele repair with a biologic graft may reduce failures by reinforcing the patient's attenuated endopelvic fascia and by covering specific defects that might be missed during site-specific rectocele repair. An alternative theory was that a biologic graft may provide a lattice for native tissue incorporation during the healing process, depending on the type of graft and the chemical makeup of the material. More recent studies, however, have not shown that the initial high cure rates seen with biologic grafts are holding up over time.

Altman et al. evaluated rectocele repair with a porcine xenograft (Pelvicol®) by primarily covering the fascial defect with the graft and not completing any type of repair under the graft[38]. Their study showed cure rates of only 72% at 1 year and 59% at 3 years, and in the patients who had failure and required more surgery, they found no evidence of the graft at all. They theorized that certain patients may have collegenases that break down a biologic graft prior to the time that it takes for incorporation and tissue in-growth into host tissues to occur, therefore losing the benefit of the graft. Similar poor anatomic cure rates have been recently reported for the anterior compartment with Pelvicol® use. Wheeler et al. reported a 50% failure rate in a prospective study evaluating the use of Pelvicol® for the anterior compartment[39].

The use of synthetic mesh grafts in rectocele repair would seem to overcome this deficit of biologic material as they are permanent in nature. Recent studies utilizing a Type I polypropylene mesh have shown improved cure rates that seem to be holding up over time, with minimal complications. Concerns, of course, with a permanent synthetic mesh are the potential complications, such as patient's tissue tolerability of a permanent graft, mesh extrusion through the epithelium, vaginal fibrosis, dyspareunia, and/or erosion of the mesh into adjacent visceral structures over time. Again, synthetic mesh use has been shown to be safe and acceptable in sacralcolpopexy, and initial studies with mesh grafts placed vaginally have shown the benefits of increased cure rates with low rates of complications (Tables 1 and 2). Most complications, such as mesh extrusion or vaginal exposure, are relatively rare and easily handled, and do not necessitate the complete removal of the mesh. These risks and others, such as dyspareunia, and the prevention of and management of these complications if they do occur, will be discussed later.

Anatomically, the placement of a graft in the posterior compartment is well suited to treat rectoceles and enteroceles; however, the apex of the vagina still needs to be supported because most patients with significant posterior wall defects also have vault prolapse. Traditionally, vaginal vault suspension treated vaginally has been approached through the posterior compartment, with utilization of the uterosacral or sacrospinous ligaments. Secondary to this, many different techniques were utilized to attach the apical portion of the graft to these ligaments or the iliococcygeus fascia to achieve vault suspension as well. However, these techniques involved complicated dissections and required advanced surgical skills, and therefore limited the overall use of vaginal mesh to treat both the posterior and apical compartments. The abdominal approach utilizing mesh sacralcolpopexy has been shown to have the highest cure rate in the literature to treat vault prolapse and has been shown to be superior to traditional vaginal approaches; however, it carries a much higher morbidity than vaginal procedures and the search for a less invasive, but as effective, treatment for vault prolapse therefore continues. One thought is that it may not be the connection of the graft to the sacrum that increases abdominal sacralcolpopexy cure rate, but rather the fact that this is the only procedure that traditionally utilized a graft attached to the vagina and that may be the reason why the failure rate is lower.

## VAGINAL MESH KITS FOR THE POSTERIOR AND APICAL COMPARTMENTS

The posterior IVS (intravaginal slingplasty) developed by Petros was the first kit to be marketed for a minimally invasive approach to treat vaginal vault prolapse. It was first described by Petros[40] as a less invasive alternative to abdominal sacralcolpopexy for the treatment of vault prolapse. It involved insertion of a polypropylene mesh tape through two small incisions in the buttock lateral and inferior to the anal orifice into the ischiorectal fossa, through the levators and iliococcygeus fascia at the level of the ischial spines on both sides, and attaching the tape to the vaginal vault. The tape was inserted through a tunneling device and aimed to suspend the vaginal apex in its natural position in a tension-free manner by creating a neoligament at the level of the cardinal ligament. Initial retrospective reports were encouraging[41] and cure rates in subsequent papers with longer follow-up still seemed to be adequate; however, complications involving the multifilament polypropylene mesh began to be reported. In a randomized controlled trial by Meschia et al. of 66 women, which compared posterior IVS with vaginal sacrospinous colpopexy, no significant difference was found in terms of prolapse recurrence[42]. Both groups had similar operating times and hospital stay. However, the posterior IVS was associated with pararectal pain (9%), pararectal abscess (3%), and vaginal vault tape extrusion (3%). Siegel et al., in a case series of 35 patients with multifilament sling, reported defective vaginal healing in 17% of cases with mesh extrusion[43]. Luck et al. more recently reported the same 17% rate of extrusion in a series of 90 patients[44]. Baessler et al. reported similar, severe, mesh-related complications, including retropubic abscess, vescicovaginal fistula, severe dyschezia, and dyspareunia necessitating sling removal[45].

The posterior IVS gives an excellent example of how mesh properties can affect outcomes. The procedure was a novel, minimally invasive, safe approach to treat vault prolapse that restored normal anatomy with initial cure rates that were very encouraging. However, the properties of a multifilament (even though still polypropylene, but not Type I) mesh caused multiple complications, including mesh extrusions, abscess formation, and rejection of the mesh tape in many patients. Ultimately, it was not the procedure itself, but the properties of the mesh that caused the complications. Additionally, many surgeons felt that a graft still needed to be placed in the posterior compartment and, therefore, were attaching the IVS tape to the top of the graft to achieve vault support and, in essence, making the first posterior compartment kits.

The Apogee procedure (Fig. 2) from American Medical Systems was the first kit to be released in the U.S. (in 2004) that treated rectocele, enterocele, and vault prolapse concomitantly with one procedure. It combined the concept of a posterior wall graft to treat rectocele and enterocele, in combination with the IVS concept of two lateral arms connected to the apex of the graft, traversing the iliococcygeus fascia and muscle to support the vaginal vault. The procedure, however, has several modifications and improvements over the IVS procedure, including utilizing a mesh graft with the arms preattached to treat both the posterior compartment and apex at the same time. Other modifications include the utilization of a macroporous, monofilament, soft polypropylene mesh (Type I) both in the graft and the lateral arms, and modifications of the needle that is passed through the ischiorectal fossa that make the needle pass safer (much smaller diameter needle) and give it the ability to get a slightly higher pass through the side wall (closer to the ischial spine), therefore achieving more apical support.

## Surgical Procedure and Relevant Anatomy: Posterior/Apical Compartment

The vaginal dissection utilized for the posterior systems involves essentially the same dissection one would use for a standard posterior repair. The vaginal epithelium is again infiltrated with a solution that contains a vasopressor agent, both for hemostasis and for hydrodissection. A thicker dissection plane should be sought to help avoid mesh complications. In standard repairs, the vaginal epithelium is left very thin; however, when a synthetic mesh is being placed, the dissection plane should be deeper because it is thought that this may help to avoid complications such as extrusion or dyspareunia. The incision should be kept as small as possible (3–4 cm), and the dissection should be tunneled up under the vaginal epithelium, out laterally to the levators up to the ischial spines, and apically to the cervix or the cuff of the vagina. Again, as in the anterior dissection, it is crucial to dissect all the way up to the cuff of the vagina so that the mesh will lie flat and not bunch up near the top of the vagina. Once the dissection is complete, two small stab incisions are made 3 cm lateral and 3 cm inferior to the anus. The curved needles are then passed laterally via the ischiorectal fossa, parallel to the rectum. As the needle is passed through the space, the surgeon's index finger is used to palpate the needle on the other side of the levators and guide it up to the level of the ischial spine (Fig. 9). The middle finger is used to deflect the rectum medially. This technique limits blind passage of the needle and minimizes the risk of bowel perforation. The needle tip is then delivered through the iliococcygeus at the level of the ischial spines and is brought out approximately 1–2 cm from the side wall and not out of the vaginal opening. This helps to maintain the correct position of the needle and ultimately the correct position of the mesh arm supporting the apex. The arms of the mesh are then attached to the needles and withdrawn through the buttock incisions. This positions the arms at the level of the ischial spines, which allows them to function as neoligaments, analogous to the cardinal ligaments. The tail of the mesh is then tailored to the vaginal length (approximately 2 cm of mesh should be left above the arms as to not shorten the vagina) and the apex of the mesh is attached to the cuff of the vagina or the posterior aspect of the cervix if the uterus is still in place. The arms of the mesh are then adjusted in a tension-free manner and the lateral aspect of the graft is attached to the pelvic side wall with two to three sutures per side down to the level of the perineal body. The mesh should be cut to fit the vaginal length and width; however, it should be left somewhat loose to allow for some mesh and tissue constriction while healing. Minimal to no vaginal epithelium should be

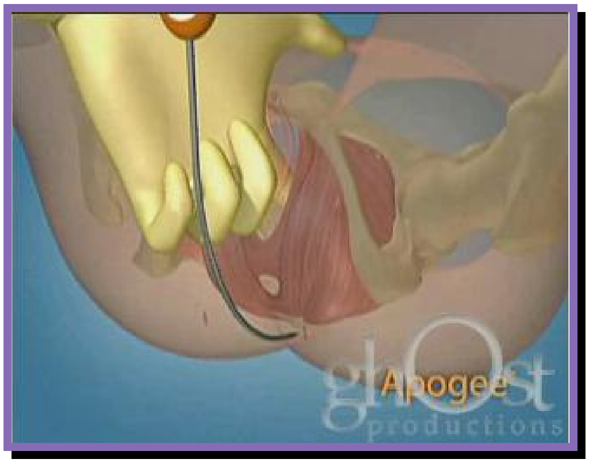

**FIGURE 9.** Apogee needle pass.

excised. The tension of the arms should be checked via a rectal exam prior to removing the plastic outer sheaths to ensure that they are not under any tension. If a tight band is felt on the rectal exam, lifting up on the arm with the finger in the rectum should loosen the arm. The plastic outer sheath is then removed, which locks the arms in place. The excess mesh is then trimmed and the buttock incisions are closed with Steri-Strips™.

The posterior Prolift procedure (Gynecare) and posterior Avaulta procedure (Bard) were also subsequently released in the U.S., and are similar to the Apogee System in the idea of treating the entire posterior compartment and apex with one surgical kit (Figs. 3 and 4). A group of nine surgeons from France formed a study group to investigate the potential for incorporating an adjustable winged delivery system into the grafted approach to overcome the inherent shortcomings in suture-fixated transvaginal grafts. This was a 2-year process that helped to combine the technologies available, and the surgeons had an inherent role in developing and perfecting the procedures. The group also tracked all operative data, including complications, during this time frame and accumulated 687 patients by 2005[46], prior to the Prolift System being released in the U.S. by 2005.

The Prolift System also utilizes a soft, macroporous, monofilament, polypropylene Type I mesh, passed through the same incisions as the Apogee System; however, the major difference is that the delivery system and the apical arms of the posterior system are designed to be passed through the sacrospinous ligaments. This may achieve a more apical support; however, there is also a concern of a higher risk of neurovascular complications (pudendal nerve injury, pain, etc.) with passage of the needles through the sacrospinous ligaments and the question that remains to be answered is: Is it actually necessary to have to do this? The IVS system was developed by Petros as a tension-free vault suspension with support at the level of the ischial spines (which is what the Apogee and posterior Avaulta Systems use) with a less invasive approach compared to the sacrospinous ligament fixation (SSLF). The vaginal apex is another 2–2.5 cm higher than the spines; however, the tape acts as a seatbelt so that the vault does not come down beyond the spines, and this has not seemed to affect true cure rates. The vaginal axis also stays in its physiologic anatomic position with this type of approach, and does not pull the vault posterior and change the axis, thus increasing the risk of rectal dysfunction or the formation of a cystocele postoperatively as has been seen with traditional SSLF.

The Bard posterior Avaulta System utilizes a collagen coating over a Type I, macroporous, polypropylene mesh that is advertised to reduce the inflammatory response during the healing phase. However, there are no clinical data to date to support this in the literature[30], and the mesh is much stiffer and more dense than either the mesh used in the Apogee System (Intepro Lite) or the Gynemesh PS (Soft Prolene) used in the Prolift System. The posterior Avaulta System has apical arms similar to Apogee and Prolift; however, it also has distal arms that attach through the perineal body via extra incisions and needle passes through the perineum (Fig. 4). There has been some concern that these passes could increase the risk of dyspareunia at the level of the perineal body, but again, there is no literature to prove or disprove this.

## VAGINAL MESH KITS — CLINICAL DATA

The newer generation vaginal mesh kits as discussed in this review were only recently released over the past 2–4 years in the U.S.; therefore, clinical data are still in their infancy. Recent studies are summarized in Table 3. Some will argue that technology is outpacing science, and industry is releasing these new techniques and "kits" prior to having adequate data. Of course, the difficulty of this is that, in a flat world, surgeons (such as those in France and other parts of the world) utilize materials to help develop new techniques, and the techniques and ideas then spread much more rapidly across the world. Other surgeons then put their "twist" on the idea or feel that they can have better results if they use a different mesh, a different way to put the mesh in place, a different technique on where and how to attach the mesh in place, or add other variations to the procedure. Therefore, there is a hodgepodge of ideas and techniques, and it becomes very difficult to study and reproduce one surgeon's technique vs. another. Industry and companies then get involved to try to commercialize these ideas, and certainly one of their primary goals is financial gain; however, they also have given us the advantage of attempting to standardize some of these techniques to obtain reproducible results globally. They have the resources to bring many surgeons and scientists together, to share ideas and techniques, and then to develop the techniques that seem to work the best together. Most of these techniques are modifications of procedures that have been performed for years, including the current mesh kits. Vaginal mesh repairs have been completed for many years and, over the years, many studies have been published (Tables 1 and 2). Over these years, the best-tolerated mesh to date has been identified (Type I, macroporous, soft polypropylene), techniques in dissection and mesh placement to minimize risks of complications have been identified, and kits have now been developed to not only standardize the techniques, but also to find the least invasive way to get the mesh in place. No surgery will ever be risk free; however, the goal in prolapse surgery certainly is to have the longest-lasting anatomic repair with minimal risks of complications, including pain, infection, bladder or bowel dysfunction, or sexual dysfunction. One does have to remember that our standard techniques, such as anterior and posterior colporrhaphy, were never studied in comparison to other techniques and our baseline "standards" are very low with these techniques, i.e., with high failure rates and high rates of dyspareunia; therefore, that is why the search is on for new techniques with better results. However, that does not give us the excuse not to study new techniques and ensure their safety and efficacy properly, with adequate scientific trials, prior to recommending their use globally. The data presented in the following section on these new vaginal mesh kits for prolapse are relatively sparse and not long term; however, more and more data are being accumulated, presented at specialty meetings, and in the process of being published to support the techniques and ultimately improve on them as well. The safety and efficacy of mesh use in pelvic surgery has been studied and proven; however, these new techniques in vaginal mesh prolapse repairs need further, more rigorous, study to help surgeons decide which is the best candidate for the procedures in order to maximize results and minimize morbidity. These are answers we still need. Companies such as Gynecare and American Medical Systems should be commended and not necessarily condemned, as both companies gathered initial data in a prospective fashion prior to mass release of their products in the U.S., and as can be seen below, are involved with current prospective multicenter trials that are still ongoing in the U.S. and worldwide. Of course, the ideal studies will be prospective randomized controlled trials conducted by independent researchers without any industry sponsorship; however, these can be very costly and difficult to achieve and one must also remember that surgical trials have one of the most difficult variables to control…the surgeon's expertise and ability to perform the actual procedure.

**TABLE 3**
**New Mesh Kits for Prolapse**

| Study | System and Mesh | No. of Patients | Follow-Up | Anatomic Cure | Comments |
|---|---|---|---|---|---|
| Moore and Miklos[49] | Perigee Intepro Type I polypropylene mesh | 77 | 1.5 Years | 93% | 6.5% Mesh extrusion, no bladder injuries, no groin pain |
| Nguyen and Burchette[50] | Perigee vs. anterior repair | 38 vs. 37 | 12 Months | 87 vs. 55% (*p* = 0.005) | 9% Dyspareunia in Perigee vs. 16% anterior repair |
| Gauruder-Burmester et al.[51] | Apogee and Perigee Type I polypropylene mesh | 72 Perigee (anterior), 48 Apogee (posterior) | 1 Year | 93% | 3% Vaginal mesh extrusion, no new onset dyspareunia |
| Rane et al.[78] | Perigee | 70 | 18–36 Months | 95.5% | 7.1% extrusion, 1.4% dys-pareunia, no groin/thigh pain |
| Fatton et al.[53] | Prolift Type I polypropylene mesh | 22 Anterior, 29 posterior, 59 both | 6 Months | 96.7% | 4.7% Mesh extrusion, 1% bladder injury, 2% hematoma |
| Altman and Falconer[57] | Prolift | 106 Anterior, 71 posterior, 71 both | Operative data only | N/A | Five bladder perforations, five rectal perforations, one bleed >1000 cc |
| Altman et al.[54] | Prolift | 123 | 3 Months | 87% Anterior, 91% posterior | 3.2% Injury (three bladder, one rectal) |
| Sola et al.[55] | Prolift | 41 | 7 Months | 91% | One extrusion, one perirectal hematoma |
| Van Raalte et al.[68] | Prolift | 46 Anterior, 28 posterior, 23 both | 19 Months | 86.6%, (96.5% Apical cure) | 0% Extrusion, 15% prolapse in untreated compartment |
| Hinoul et al.[69] | Prolift-Anterior | 48 | | 95.8% | 10.5% Extrusion, 15% dyspareunia |

## Perigee and Apogee Clinical Data

The Perigee and Apogee procedures were the first mesh "kit" procedures to be released in the U.S. and, therefore, they do lead the way in clinical data to support these techniques. Moore et al. reported on the safety, efficacy, and short-term follow-up in the first prospective multicenter trial on Perigee for cystocele repair in the U.S.[48]. Women with symptomatic Stage II or greater cystoceles were treated with the Perigee procedure at eight centers across the U.S. Additional procedures for defects in other compartments and/or incontinence were completed as necessary. Six-month follow-up on 84 patients revealed a 91.4% cure rate (Stage I or 0) for the anterior compartment and a mesh extrusion rate of 7.7%. Of those patients with mesh extrusions, 37.5% healed with conservative or in-office treatment, and the others had the mesh extrusion treated in the operating room with simple excision of the exposed mesh and primary closure of the defect. There were no reported infections of the mesh, nor any long-term sequalae of the mesh extrusions, nor did any graft have to be removed in its entirety because of mesh infection or extrusion. There were no bladder injuries at the time of surgery and no mesh erosions into the bladder reported on follow-up. There were no reports of any persistent or long-term pelvic, groin, or thigh pain (although one patient did have the graft removed at 1 month secondary to vaginal pain that was not resolving). Quality of life (QOL) questionnaires revealed statistically significant improvements, including the Prolapse and Incontinence Sexual Function Questionnaire (PISQ).

Moore and Miklos[49] reported 1.5-year retrospective data on a series of 77 women who underwent the Perigee procedure at their center for symptomatic Stage II or greater cystocele (40.2% of patients had a failed previous cystocele repair). Additional reconstructive and anti-incontinence procedures were performed as needed. Eight patients also had an Apogee placed in the posterior compartment simultaneously and 12 other patients had posterior wall mesh (Gynemesh) placed for rectocele treatment. There were no bladder injuries passing any of the needles. There was one midline cystotomy reported on dissection that was repaired and the Perigee still placed without sequalae. Average blood loss was 77 ml (range: 10–400 ml). Two patients had intraoperative bleeds that formed hematomas under the incision; however, these were evacuated and controlled during the initial surgery and no patients had to be taken back to the operating room for bleeding or pain. One of the patients with an intraoperative bleed (400 cc) did have a transfusion of 1 unit of packed red blood cells postoperatively. This was the only transfusion given during the study. Cure rate at 18.2 months (Ba $\leq$ 1.0) was 93.5%. Mean vaginal length did not change significantly from pre- to post-op. No patient complained of long-term vaginal or pelvic pain, and no mesh had to be revised or removed because of dyspareunia. Mesh extrusion rate was 6.5% (5/77) with four of the five patients requiring local excision of the exposed mesh only in the operating room. All healed without further sequalae. All exposures were less than 1 cm in size, and no patients had evidence of infection or rejection of the mesh. Seventy-three percent of patients that suffered from urgency symptoms preoperatively had resolution of these symptoms and 3.8% developed *de novo* urge symptoms postoperatively.

Nguyen and Burchette[50] recently reported on the first randomized controlled trial (Level I evidence) of a vaginal mesh kit procedure with the Perigee procedure compared against traditional anterior repair. Seventy-six patients were randomly assigned to undergo the Perigee procedure or anterior repair for symptomatic Stage II or greater cystocele. One surgeon performed all procedures and was blinded to the randomization. Concomitant procedures for prolapse and incontinence were permitted. There was no difference in blood loss, blood transfusion rates, or urinary tract infections postoperatively. Post-op unilateral groin pain was present in one Perigee patient, which resolved with oral analgesics and was resolved by the 8-week post-op visit. There were two patients (5%) that suffered 2–3 mm asymptomatic mesh extrusions that were both treated in the office with simple excision and vaginal estrogen, and healed without sequalae. There was no mesh infection or rejection noted. At 1-year follow-up, optimal and satisfactory anterior vaginal support was significantly higher in the Perigee group (87%) compared to the colporrhaphy group (55%). *De novo* dyspareunia occurred in 16% of the colporrhaphy group vs. 9% of the mesh group, which was not statistically different. Their conclusion was that repair with Perigee offers lower failure rate than anterior repair at 1 year with no increase in sexual dysfunction.

Gauruder-Burmester et al.[51] reported on 120 patients receiving Apogee and/or Perigee for recurrent prolapse with 1-year follow-up, with a special focus on mesh extrusion and dyspareunia. All patients had recurrent Stage III or greater prolapse. Forty percent of patients had an Apogee procedure for rectocele and vault prolapse, and 60% had Perigee for recurrent cystocele. No patient had both placed at the same time. No intraoperative complications were reported. Mesh extrusion occurred in four patients (3%) and involved anterior mesh placement alone. No rectal erosions were reported. No mesh infections were reported. At 1 year, 93% of patients were cured of prolapse (Stage I or less). Prolapse occurred in eight women and all failures were in the anterior compartment. No dyspareunia was associated with the repair and, in fact, prolapse-associated dyspareunia resolved in all 15 patients who reported symptoms prior to surgery.

## Prolift Clinical Data

Cosson et al. were part of the initial group of TVM (transvaginal mesh, or sometimes also referred to as tension-free vaginal mesh) surgeons in France who worked on developing the Prolift System, and initially reported on a group of 684 women who underwent transvaginal mesh placement using trocars passed through the obturator (anterior) and ischiorectal space (posterior and apical) at the ICS Meeting in Montreal, Canada in 2005[46]. They followed this study with the first retrospective report on 90 women who underwent the actual Prolift kit procedure in a multicenter trial in France[52]. The mean age in this group of 90 patients was 65.3 and 72 of them underwent concurrent hysterectomy. Cure rate at 1 year was noted to be 82% (18% failure rate). Vaginal mesh extrusion rate was 6.7%, with risk of mesh extrusion increased with anterior Prolift, concurrent hysterectomy, and using T-shaped incision at the cuff with hysterectomy. *De novo* dyspareunia was noted to be 4.9%.

Fatton et al. published one of the first retrospective, multicenter series that reported on initial operative and short-term follow-up data on 106 patients who underwent the Prolift procedure. All patients had a Stage III (at the hymen) or Stage IV (beyond the hymen) prolapse[53]. Total mesh was used in 59 patients (53.6%), an isolated anterior mesh in 22 patients (20%), and an isolated posterior mesh in 29 patients (26.4%). One bladder injury was reported that was sutured at surgery and two hematomas reportedly required secondary surgical management. At 3 months, all 106 patients were available for follow-up. Mesh exposure occurred in five patients (4.7%), two of them requiring surgical management. Granuloma without exposure occurred in three patients (2.8%). Failure rate (recurrent prolapse, even asymptomatic or low-grade symptomatic prolapse) was 4.7%. No sexual function data were reported due to the short follow-up.

The Nordic TVM group published the first prospective study to date on the short-term outcomes of transvaginal repair of prolapse using Prolift[54]. They are conducting a 3-year prospective multicenter trial in 28 centers and have published on the initial safety and early efficacy of the procedure with 2-month follow-up of 123 cases. There were visceral injuries reported in 3.2% of the cases (three bladder injuries and one rectal injury) and only two mesh exposures reported at 2-month follow-up. Postoperative cure (POP-Q Stage 0 or I) was 87% after anterior Prolift, 91% after posterior Prolift, and 88% after total Prolift. They also did a macroscopic assessment of the vaginal epithelium and noted an increase of mild-moderate granuloma formation in the operated areas, but no cases of serious adverse tissue reactions related to the mesh. All quality of life scores improved at the 2-month visit. There were no serious adverse events attributed to the polypropylene mesh.

Van Raalte et al. recently reported on 97 patients who underwent the Prolift procedure with a median follow-up of 19 months[68]. Prolift procedures included 46 anterior, 26 posterior, and 23 total (both anterior and posterior). Intraoperative complications included four cystotomies (8.6%) and one ureteral obstruction. Overall cure rate at 1 year or greater was 86.6% for all compartments. Failure in the same site that mesh had been placed was lower at 10%; however, they did find that prolapse occurred in 15.5% in an untreated compartment. Apical cure was 96.5% and reoperation rate for prolapse was low at 4.3%, which they stated is comparable to abdominal sacralcolpopexy at 4.4%. They reported no long-term complications and no mesh extrusions. Dyspareunia rates were not reported. They concluded that the potential for improved surgical outcomes will be achieved only through modifications of techniques, longer-term follow-up, and proper patient selection.

Sola et al. and Hinoul et al. have the only other published series in the literature regarding the Prolift procedure[55,69]. Sola et al. retrospectively reported on 41 patients who underwent the Prolift procedure, with an average of 7 months follow-up (range: 2–12 months). One perirectal hematoma occurred postoperatively. Four failures were noted with a cure rate of 91.3%. Only one mesh extrusion occurred (2.4%). Hinoul et al. most recently prospectively reported on 48 patients who underwent anterior Prolift. They reported a 95.8% cure rate with 10.4% rate of mesh extrusion. In nine of 29 (31%) sexually active patients, dyspareunia due to prolapse was present prior to surgery and disappeared in all. *De novo* dyspareunia, however, did occur in 15% of patients[69].

### Avaulta Clinical Data

To date there are no published series on the Avaulta procedure.

## COMPLICATIONS PUBLISHED IN THE LITERATURE

Secondary to the "blind" passage of needles involved in the transvaginal placement of mesh with kits, an in-depth knowledge of pelvic anatomy is crucial to help avoid complications that can occur with these procedures. An inadequate dissection or deviation off the appropriate course of the needle can result in complications and injury. The shapes of the needles themselves may also have a role in the prevention of injury, as the different kits do differ somewhat in needle size and shape, especially with the anterior compartment. Surgeons that are familiar and experienced with the procedures may be comfortable with any shape and size needle; however, the size and shape of the needles, especially with inexperienced surgeons, may have a role in the rate of complications. The Perigee procedure utilizes a helical-shaped needle for both the bladder neck and the more apical pass near the ischial spine, which limits the depth and prevents the user from passing the needle beyond the ischial spine, therefore potentially decreasing risk of injury. The anterior Prolift procedure uses the same long, curved needle that is used for all passes (both anterior passes and the posterior pass) and therefore the depth is not controlled by the shape of the needle. The posterior Apogee needle is very similar in shape to both the Prolift and Avaulta needles; however, as stated earlier, the Prolift needle is designed to be passed through the sacrospinous ligament, which is a more dangerous pass secondary to the proximity of the pudendal neurovascular bundle to the ligament itself. The posterior Avaulta and Apogee procedure needles are designed to be passed through the iliococcygeal fascia just distal to the ischial spine, which is a safer distance from the pudendal bundle and may decrease risk of injury in this location. These points were demonstrated in two cadaver studies that showed the bladder as well as the rectum, and significant vascular structures, such as branches of the internal pudendal vessels, were shown to be in very close proximity of the Prolift kit needles when the needles were placed using the recommended guidelines[47,56]. This may demonstrate that variations in the individual kits themselves, including mesh size, mesh composition, size and shape of needles, and recommended location of the passage of the needles, may also contribute to different complications. The following is a summary of the complications of these procedures that have been published in the literature to date and may demonstrate how some of these injuries or complications could be related to the shape of the needles, the location of the particular procedure's needle path (i.e., sacrospinous ligament or iliococcygeus fascia), and/or surgeon's experience or competency. As we know, no surgery is immune to complications for these various reasons.

In a published series of 248 patients who underwent the Prolift procedure, Altman and Falconer reported on the frequency and type of perioperative complications that occurred with the procedure[57]. During the study, 106 patients had anterior Prolift, 71 posterior Prolift, and 71 total Prolift (anterior and posterior). It may not be surprising, given the above cadaver studies showing the proximity of the Prolift needles to visceral and vascular structures, that 11 major complications (4.4%) were reported (five bladder perforations, five rectal perforations, and one major vascular bleed of more than 1000 cc). Their conclusion was that perioperative serious complications are uncommon; however, special care should be used to detect possible visceral injuries at the time of surgery.

There also have been other published reports of acute hemorrhage following anterior Prolift placement, as well as an expanding pararectal hematoma that was diagnosed 24 h after placement of a total Prolift. LaSala and Schimpf reported on two cases of postoperative hematomas after anterior Prolift[58]. Neither needed drainage, however, one did require a blood transfusion and the other needed intravenous antibiotics to treat an infected hematoma. Gangam and Kanee[59] reported on a case of acute bleeding in the recovery room of a patient following anterior Prolift that caused her to become hypotensive and be taken back to the operating room. A hematoma was found under the anterior vaginal wall incision and evacuated; however, diagnostic laparoscopy revealed 200 cc of blood in the cul de sac,

400 cc in the retropubic space, and bilateral hematomas seen in the pararectal spaces extending to the infundibulopelvic ligaments. No acute arterial bleeding was seen and she recovered without needing embolization, but did require transfusion of 3 units of packed red blood cells. Touboul et al. and Mokrzycki et al. both published separate case reports[60,61] on life-threatening pelvic hemorrhage following anterior Prolift placement (inferior/apical anterior needle passage). Both cases were treated successfully with pelvic artery embolization and packing.

To date, there are no case reports of serious acute hemorrhage published with the anterior Perigee procedure. As stated earlier, this may be secondary to the fact that each needle in the Perigee and Apogee kits is specifically designed for each pass and, therefore, there are three different-shaped needles (Figs. 1 and 2). The helical shape of the Perigee needle was designed to limit the depth of the needle pass, which may help to reduce risk of major vascular complications. However, the system is not immune to complications as there has been one case report of a vesicovaginal fistula following a Perigee procedure[62].

Dyspareunia requiring removal of the posterior mesh arm from the sacrospinous ligament following posterior Prolift has also been reported[63]. The patient was treated conservatively at first with physical therapy, anti-inflammatories, vaginal estrogen, and local injections; however, her pain did not improve over a 30-month period. On exam, the arms of the posterior mesh could be palpated as they passed through the midportion of the ligament and were subjectively tight and, when palpated, reproduced the patient's pain. The mesh arms were resected and removed from the ligaments and the patient's dyspareunia completely resolved. At our center in Atlanta, we also had a patient that presented with similar symptoms following posterior Prolift with passage of the arms through the sacrospinous ligaments. The patient was a 38-year-old female who initially had a vaginal hysterectomy with posterior Prolift for reported Stage II prolapse. Postoperatively, she suffered acute pain radiating down her left buttock and posterior thigh, and vaginal pain shooting down the inner thigh, which made it difficult for the patient to sit down or walk. The pain did not resolve with conservative management, nor could the patient return to work; therefore, the original surgeons attempted resection of the mesh on two occasions. She also had pelvic floor physical therapy and trigger point injections into the region, but still had minimal improvement. On exam, the posterior arm could still be palpated on rectal examination, under tension, going through the sacrospinous ligament, and her pain was reproduced when this area was palpated. Secondary to this, we used a posterior wall approach to isolate the remaining portion of the arm that was still present going through the ligament and removed it in its entirety, including the portion going through the sacrospinous ligament. Following this and further pelvic floor physical therapy, the patient has had resolution of about 75% of her pain and continues to improve slowly.

These cases may demonstrate the fact that pudendal nerve injury or entrapment is a risk with passage of the mesh arm completely through the ligament from a posterior (below-up) approach vs. passage of the arm through the iliococcygeus fascia at the level of the ischial spine as described by the original IVS procedure and the approach that the Apogee and Avaulta procedures have adopted as well.

Two centers recently reported on complications due to vaginal mesh kits that presented at their individual centers in separate reports[70,71]. Margulies et al. reported 13 cases of complications that presented at their tertiary referral center following Apogee or Perigee placement that were completed at other centers. Ten patients had symptomatic mesh exposure, one had an exposure with pelvic abscess, and two had pain syndrome without mesh exposure. Additionally, one patient had mesh erosion into the rectum with fistula and one a vesicovaginal fistula. The primary complaint was pain in nine of 13 (69%). Patients required a median of two surgeries per patient to treat these complications. They described their techniques in managing these complications and did find mesh folding in 69% of patients and concluded that this may be a major factor causing extrusions and/or pain[70]. Ridgeway et al. reported on 19 patients that presented to their tertiary care facility with complications from vaginal mesh kits that required surgical intervention[71]. The majority of the cases involved Prolift (17/19) and mesh extrusion (12/19). Additional symptoms were pain in lower extremities or vagina (6/19), dyspareunia (6/19), fistula (3/19), and recurrent prolapse (8/19). They also described the surgical techniques of managing these complications and found that 93% of patients were satisfied with the surgical treatment of their complication. They were also careful to state that no inference should be made on the overall incidence of mesh complications secondary to the fact that the overall number of procedures that resulted in the complications was unknown.

These reports do emphasize the concern that complications can occur following mesh placement and, in some cases, can be serious and affect quality of life greatly, including fistula, pain, or dyspareunia. The overall percentage rate of complications cannot be truly calculated and is difficult to ascertain from these types of reports secondary to the fact that the total number of procedures completed is unknown (i.e., did 10 complications occur in 100 patients or 1000?). It is clear, however, that many surgeons who are completing these procedures may not be experienced enough or have the skills to take care of the complications; therefore, the patients are then sent to specialists who seem to be somewhat successful in managing the complications. It does, however, lead to the question: Are these complications occurring because inexperienced or unqualified surgeons are completing the procedures in the first place? Or, is it secondary to the fact that the overall numbers of patients being treated for prolapse have increased a great deal because these kits and treatments are now available to generalists and not just specialists? Therefore, more prolapse is being treated and more individual incidences of complications secondary to the prolapse treatment are being seen or sent to the specialist that may not have performed the procedures? And are these risks balanced by the increase in overall cure rates seen to date in the literature when a mesh is added to treat prolapse? Of course, these are all issues that need to be studied further in the future with well-controlled trials. One also has to recall that traditional surgery for prolapse also carries inherent risks of not only complications, but also a higher risk for recurrent prolapse, a risk that cannot be discounted.

Dyspareunia is one of the complications that has been receiving a great deal of attention in the literature and at scientific meetings. Interestingly though, there have been several recent reports that have shown that the overall risk of dyspareunia, when carefully studied and when the procedure is done by experts, is actually lower than traditional surgery for prolapse. Lowman et al. showed the overall risk of dyspareunia for the Prolift procedure to be 16.7%[72]. Although, at first glance, this rate does look high, they compared it to historical control studies and found that even with traditional vaginal or abdominal repair for prolapse, dyspareunia rates are in the range of 14.5–36.1%. Therefore, they concluded that the Prolift procedure has comparable rates of dyspareunia. Nguyen and Burchette, in a prospective randomized study comparing Perigee to anterior repair, showed dyspareunia rates to be higher in the anterior repair group (16 vs. 9% with Perigee), although statistically this was not significant[50]. Nieminen et al. showed a statistically lower incidence of dyspareunia compared to anterior repair in another prospective randomized trial comparing anterior wall mesh to traditional repair without mesh[73]. Finally, Gauruder-Burmester et al. completed an in-depth sexual function analysis on 120 women who underwent prolapse repair with Apogee and/or Perigee, and found that none of the 120 sexually active patients had a sexual problem attributable to the mesh repair[74]. Forty patients did suffer from a sexual disturbance; however, in none of these cases was the disturbance related to the repair. They concluded that their results suggest that many investigators prematurely attribute sexual problems to a urogynecologic operation without adequate evidence in the form of comprehensive sexual medical examinations of their patients.

## AVOIDING AND TREATING COMPLICATIONS OF MESH KITS

### Proper Patient Selection

Proper patient selection is the best way to avoid unnecessary complications. There are no randomized controlled trials published for guidance on who the correct patients are on which to utilize mesh and mesh kits, nor will there be in the near future. However, at this point in time, it is our belief that vaginal mesh should not be used in all patients across the board that present with prolapse. A 32-year-old female with a primary Stage I or early Stage II cystocele or rectocele probably should not have vaginal mesh placed for treatment. Mesh should be used selectively in patients that will benefit from its use and where the benefits outweigh the potential risks. Patients must also be fully informed and educated on the potential benefits of graft use and also the potential risks. Currently, our recommendations for its use are in patients with recurrent prolapse and failed previous procedures, larger prolapse (Stage III or greater), and in elderly patients or those with poor tissue quality (postmenopausal or older) (Table 4). Some surgeons consider patients with diabetes or collagen-vascular disorders (such as lupus, etc.) as having relative contraindications to mesh use; however, we have not seen an increase in complications in these types of patients and, given their tissue quality, they are patients that have a high risk of failure and would most likely benefit from the utilization of mesh grafts. In younger, sexually active patients (i.e., <50–60 years old) with more extensive primary prolapse, it is our practice to utilize mesh in their repair at the apex only, i.e., with laparoscopic sacralcolpopexy. This avoids mesh placement in the distal vagina; however, the patient still benefits from the use of mesh in the repair at the apex of the vagina and it helps to support other portions of the repair that we do not use mesh on directly. We feel this may help to decrease risk of dyspareunia; however, it does not eliminate it, as there is a baseline risk of dyspareunia with sacralcolpopexy as well.

**TABLE 4**
**Considerations for Mesh Kit Use**

- Recurrent prolapse
- As patient gets older and prolapse gets larger
- Advanced prolapse (Stage III or IV)
- Postmenopausal
- Decreasing estrogen status
- Poor tissue quality

## Mesh Extrusions and Exposure

It is clear that there has been a learning curve with the use of mesh vaginally, both in the characteristics of the mesh itself and the surgical techniques of the placement. Over the years, the complications, such as mesh extrusion or vaginal exposure, have decreased; however, they are not down to zero and, therefore, one must be ready to handle these exposures when they do occur. As stated earlier, these extrusions are typically not major complications. When the proper mesh is used (a Type I, soft, macroporous, monofilament polypropylene), the exposures are typically small and easily handled. What we have learned over the recent years, though, is that proper surgical technique and the skills of the surgeon are crucial in minimizing risks of complications, including mesh exposure (Table 5). The incision should be kept to the minimal size possible and should not be taken all the way up to the cuff on either the anterior or posterior wall. The dissection can be "tunneled" under the vaginal epithelium with the use of retractors and the incision does not need to include the entire vaginal wall. This concept is very simple: the smaller the size of the incision, the less risk there is of a healing defect or mesh exposure through the incision. If hysterectomy is indicated, the cuff should be closed separately and the incisions should not be a "T" shape at the cuff, as this has been shown to increase risk of mesh extrusion at the cuff[64]. Two to 3 cm of vaginal epithelium should be left between the incisions. A thicker dissection plane should also be used as this will help to "pad" the mesh and minimize risk of mesh extrusion through a thin vaginal epithelium. Minimal to no vaginal epithelium should be excised after the repair and prior to the closure, as the epithelium will retract 10–15% when exposed to the mesh. Therefore, if a large amount of epithelium is excised, this will increase risk of mesh extrusion as the patient heals. The vaginal epithelium should be well estrogenized both pre- and postoperatively with estrogen cream if necessary.

**TABLE 5**
**Minimizing Risks of Mesh Extrusion**

- Keep incisions as small as possible
- Type I soft mesh only
- No “T” incisions with hysterectomy
- Hydrodissection
- Maintain thick dissection plane
- Pre- and post-op estrogen
- Excise minimal vaginal epithelium

## Management of Mesh Extrusions

If mesh extrusions or exposures occur, they are typically handled very easily. If a true, uncoated, Type I mesh is used, the graft typically is not infected, nor will the entire graft need to be removed. There may be a mild, low-grade, vaginal infection present; therefore, most surgeons advocate the use of a vaginal antibiotic for 3–7 days (such as clindamycin or metronidazole cream) prior to treatment. If it is a small extrusion, many of these will actually heal with conservative treatment of vaginal antibiotics, followed with daily vaginal estrogen cream for 2–4 weeks. If it does not appear to be healing or re-epithelializing at this point in time, the exposed mesh will need to be excised. In many cases, this can be completed in the office with just excision of the exposed mesh under a local anesthetic. The exposed mesh should be excised, the edges of the vaginal epithelium undermined, freshened, and then closed primarily with interrupted absorbable sutures.

## Dyspareunia and/or Vaginal Pain

As with any vaginal surgical procedures, there is a risk of dyspareunia or vaginal pain when mesh is used. What many critics of these mesh procedures fail to understand is that risk of dyspareunia with many of our traditional repairs is very high. Traditional posterior repairs using fascial or levator plications can have rates of dyspareunia of at least 15% and in many series it is substantially higher[32]. Weber et al., in a report from the Cleveland Clinic, reported a 25% rate of dyspareunia with traditional posterior repair and when combined with Burch colposuspension, the rate of dyspareunia was 38%[65]. In the studies published to date, again using a proper Type I mesh, the rates of dyspareunia do not appear to be higher than many reports of traditional repairs and in some cases are lower. Theoretically, the risk of pain with intercourse should actually be lower with a graft use, as we are not plicating tissue together or pulling the levator muscles together in the midline like we do with a traditional repair. The graft goes from side wall to side wall and should not put any tension on the levators or the ligamentous attachments. To minimize the risk of dyspareunia with the use of vaginal mesh kits, no tension should be placed on the mesh arms at all. Prior to completion of the procedure, the vagina and rectum should be checked, and the lateral arms of all grafts used should be checked to ensure they are not “tight” or under tension. If they are, they need to be loosened prior to the patient leaving the operating room. Again, this is easily accomplished with the Apogee/Perigee procedures because the plastic outer sheaths should not be taken off the arms until they are checked for tension. If they are found to be under tension, they can be loosened easily prior to taking off the plastic sheaths, which will then lock them in place. Also, a thicker dissection should be used in order to have a “pad” of tissue above the mesh that cannot be palpated by the patient or the partner, and the mesh should lie flat with no tension and not be bunched up at all, especially near the apex. Minimal vaginal epithelium should be excised and the vagina should be well estrogenized both pre- and postoperatively (Table 6).

**TABLE 6**
**Minimizing Risks of Dyspareunia**

- Use tips to minimize risks of extrusion
- Type I, soft, macroporous mesh
- Minimize mesh load
- No tension on mesh or mesh arms
- Ensure mesh lies flat, no "bunching" at apex
- Maintain proper estrogenization

## Management of Dyspareunia and/or Vaginal Pain

Postoperative vaginal or buttock/leg pain should subside rather quickly following surgery. If the mesh arm has been placed through the sacrospinous ligament and the patient shows signs of a pudendal neuropathy (vaginal pain, buttock pain shooting down the back of the leg) and this does not resolve quickly, the mesh arms should be removed from the ligaments promptly to help avoid creation of a chronic nerve injury and a chronic pain scenario. This also holds true for severe groin pain following a transobturator needle passage. If the pain is not severe or mild to moderate dyspareunia occurs and does not seem to be improving with healing and time, then a conservative approach can be utilized as an initial treatment approach. Pelvic floor physical therapy with soft tissue techniques on the levators, biofeedback, electrical stimulation, and other techniques are typically very successful and may help to avoid operative intervention. Additionally, anti-inflammatory medications as well as trigger point injections vaginally using a steroid can be very helpful. If there is no improvement and if a "tight" band of mesh can be palpated in the vagina (typically where the arms are penetrating the side walls or the ligaments), these will need to be released. Typically, the entire mesh does not need to be removed, just the arm exposed that is under tension, and this arm or mesh tension needs to be cut and released. If the pain is in the midline of the vagina and not laterally out at the levators, and the mesh can be palpated through the vagina or the mesh is found to be folded or bunched up, the mesh will need to be removed in this region. Even after removal, physical therapy can continue to be very helpful (Table 7).

**TABLE 7**
**Management of Dyspareunia**

- Conservative measures
  - Pelvic floor physical therapy
  - Anti-inflammatory agents
  - Vaginal estrogen
  - Trigger point injections (steroid with anesthetic)
- Surgical intervention
  - Release any tension felt on mesh arms
  - Remove any "bunched up" mesh
  - Typically entire graft does not need removal

## CONCLUSION

Pelvic floor prolapse is a significant quality of life issue for many women in the world and in the past 10 years, more and more focus has been given to the treatment of this condition as the fields of Urogynecology, Female Urology, and Reconstructive Pelvic Surgery continue to grow. Traditional repairs have an unacceptable high rate of failure and are not immune to many of the same complications that vaginal repair with grafts are commonly criticized for. Over these past 10 years, graft use in the field of prolapse surgery has been investigated as a means to achieve a higher cure rate and ultimately gives women better long-term anatomic results following prolapse surgery. Of course, as with any surgery, the benefits have to be weighed against the risks and in prolapse surgery that includes risk of failure or recurrence of the original defect, sexual dysfunction, infection or rejection of any graft or sutures utilized, pain, and/or visceral dysfunction. Synthetic grafts used vaginally have been shown to have higher cure rates than traditional vaginal repairs without grafts and Type I, soft, macroporous, monofilament, polypropylene mesh seems to be the best-tolerated material to date. Of course, complications related to mesh use specifically, such as mesh extrusion or other potential mesh-specific complications, have to be balanced against the benefit of increased cure. Synthetic grafts have clearly established an important role in the surgical treatment of SUI and the abdominal repair of prolapse; however, their specific role in vaginal prolapse repairs still needs to be defined[75].

More recently, vaginal mesh kits have been developed in an attempt to help standardize the techniques of graft placement and make the approach less invasive. Companies have commercialized these techniques into procedural “kits”; however, it should be realized that many of the best techniques from pelvic surgeons throughout the world were brought together to help formulate these standardized kits so that more women can be properly treated globally. These techniques are really just modifications of techniques that were already being used for many years. Initial studies are very encouraging, showing high cure rates with minimal complications when properly used. These procedures are in their infancy and studies need to continue to evaluate cure rates, complications, and long-term benefits prior to making universal recommendations on their use. However, it should be noted that the studies cited in this review were completed by expert surgeons with extensive experience with graft use and knowledge of female pelvic anatomy. When used by an experienced and advanced pelvic surgeon with an in-depth knowledge of female pelvic anatomy in the proper clinical situation with appropriate patient selection, the benefits of graft use do seem to outweigh the risks. Complications still do occur, even in the most experienced surgeon’s hands, but again these seem to be minimal when experienced surgeons utilize proper techniques. However, one of the problems that does seem to be emerging is the thought that, just because these procedures are now being produced in “kits”, they can be completed by *any* surgeon. This is not true, as these are still advanced techniques that require advanced pelvic surgery skills and, therefore, it is up to surgeons to also understand this and the limitations of these procedures. That is a key element of the success of any surgery, i.e., not only correct patient selection, but also correct surgeon selection. More studies need to be completed prior to the recommendations of general use of these kits in all prolapse patients[76]. We need to have further investigation on proper patient selection, we must continue research on graft composition, and we must continue to develop techniques to minimize complications of needle passage or mesh placement. Even after we gain this knowledge, it will still require advanced surgical skills to complete these types of surgeries, and to help minimize complications and maximize results. Only surgeons who are properly trained and have proper experience should complete these repairs.
